# Bibliographical

**Published:** 1872-12

**Authors:** 


### EDITORIAL AND MISCELLANEOUS.

### BIBLIOGRAPHICAL.

*General and Differential Diagnosis of Ovarian Tumors, with Special Reference to the Operation of Ovariotomy and Occasional Pathological and Therapeutical Considerations.* By WASHINGTON L. ATLEE, M.D., of Philadelphia; with thirty-nine illustrations. J. B. Lippincott & Co., Philadelphia, Publishers.

We have received this work from the publishers, and it is offered to the medical profession by its distinguished author, and one of the most, if not the most skillful ovariotomist in the United States, in response to long and repeated calls made by numerous friends throughout the entire country. The materials are drawn from a daily record of his observations, and the book professes to be only a transcript from the author's own clinical experience, recorded at the moment of examination, and comprises the result of observations continuously pursued since the year 1843. He has written only for those who have not traveled over so large a field as himself, and has intentionally revealed his errors in order to illustrate his progressive advancement of knowledge in the diagnosis of abdominal tumors, and to show that the observer in this, as in other forms of disease, must be educated by repeated observations before he can become qualified as an expert. It is suggested, properly, that as ovariotomy has now fairly passed through its probationary career and taken a position among the established operations of surgery, a Treatise on the General and Differential Diagnosis of Abdominal Tumors, is not untimely. This volume, we are informed, precedes another (to be issued as promptly as the engagements of the author will permit—and we hope at no distant period—) on the "Surgical Treatment of Ovarian Tumors."

A chapter on the pathology of the principal varieties of cysts of the ovary is incorporated from the pen of J. Ewing Mears, M.D., of Philadelphia, and another from Thomas M. Drysdale, M.D., of Philadelphia, containing an account of the author's observations on the physical, chemical and microscopical characteristics of dropsical fluids, principally ovarian, and with special reference to diagnosis, the diagnostic value of dropsical fluids being so great in the estimation of the principal author as to justify, in doubtful cases, a resort to paracentesis, simply as a means of diagnosis. Some sixty pages of the work are devoted to the General Diagnosis of Ovarian Tumors, while the remaining pages (over four hundred) are occupied with elaborate discussions of Differential Diagnosis, including chapters on Ascites, Peritoneal Cysts, or Cysts of the Broad Ligament, Other Cystic Tumors of the Abdomen, Dermoid Tumors, Pregnancy, Pregnancy Co-existing with Ovarian Dropsy, Tumors of the Uterus, Retroversion of the Uterus, Retention of the Menstrual Fluid, Dropsy of the Amnion, Enlargement of the Abdominal Viscera, Distention of the Urinary Bladder, Accumulation of Fæces in the Intestines, Accumulation of Gas in the Intestines, Muscular Derangement of the Abdominal Parietes, Inflammatory Deposits in the Pelvic and Abdominal Cavities, Pelvic Tumor and Abscess, Spinal Curvature, Lumbar or Psoas Abscess, Malignant Tumors of the Abdomen, Rectocele and Cystocele, Hypertrophy of the Abdominal Walls, Adhesions, Pathology of Cystic Tumors of the Ovary, Dropsical Fluids of the Abdomen, their Physical Properties, Chemical Analysis, Microscopic Appearance and Diagnostic Value, based on the examination of several hundred specimens. The work concludes with some general remarks bearing upon the difficulties of diagnosis and the absolute necessity of experience to constitute an expert in any disease of difficulty, from which we extract the following: "However obscure a case might seem to the inexperienced, or however deceptive to the careless observer, it would be plain to the expert. It is so in all classes of disease; and hence, as we grow old in the profession, particularly in a large and dense population, specialties necessarily force themselves upon us. The young physician, who usually starts out as a general practitioner, attending to all branches of medicine and surgery, will find that as he accumulates experience, his success in the treatment of certain diseases will be greater than it is in others, and that his reputation will be in proportion to his success; and as a consequence his just fame for the treatment of a certain class of diseases, will, in a large city, attract so great a number of patients that in self-defense he will have to abandon other branches of his profession, as his time is monopolized by a special class of sufferers. In this way, and without any intention on his part, originates the true and well educated specialist, having a reputation based upon ample observation and experience. The opinion of such a one is properly deferred to as authoritative by his professional brethren. * * * I am sure that ovarian tumors can be recognized and distinguished from other abdominal enlargements as readily as can the several diseases of the brain, or of the chest, or of the heart, or of the nervous system; and although errors of diagnosis may occasionally occur in the former, they are not more frequent than in the latter affections."

It is unnecessary for us to commend such a work as this, as from the character of the author and this exhibit of the contents, no medical man at all interested in this subject, would fail to understand its value.

We have received from the same publishers, "Injuries of Nerves and their Consequences," by S. Weir Mitchell, M.D., "Contagious Diseases, with an account of the Primary Syphilitic Poison and of its communicability," by John Morgan, M. D., University of Dublin, Fellow of the Royal College of Surgeons, Ireland, etc., etc., " Hand Book of Compound Medicines, or the Prescribers and Dispenser's Vade-Mecum," by Arnold J. Cooley, " Angular Curvature of the Spine, with the Correct Principles of Treatment," by Benjamin Lee, M.D., which will be critically noticed in the next number, it having been found impracticable to do so in this. We have also numbers four and five of " Spectrum Analysis Explained and Spectrum Analysis Discoveries," illustrating its uses to science and showing its application in microscopical research, and to discoveries of the physical constitution and movements of the heavenly bodies, from the works of Schellen, Young, Roscoe, Lockyer, Higgins and others.

